# Cold Philosophy

**Published:** 1879-11

**Authors:** 


### COLD PHILOSOPHY.

When Patrick was asked what he would take to climb a steeple one frosty morning, "I'll take a cold, yer honor, Sawney, who stood by, said he would take a dollar. That is about the nearest description I have seen in print as to the locality best adapted for taking a cold, but that was a falsity, not a fact. The seeds of a million deaths of the beautiful, the honored and the good, will be sown this year by indifference to the statement I am going to make in reference to the time and manner of taking colds. I will not now perplex the reader with a disquisition on the physiology of colds, but will simply bring to mind what any reader will recognize as an old but forgotten acquaintance.

*The* TIME *for taking cold, is after your exercise; the* PLACE *is in your own house, or office, or counting-room.*

It is not the act of exercise which gives the cold, but it is the getting cool too quick after exercising. For example, you walk very fast to get to the railroad station, or to the ferry, or to catch an omnibus, or to make time for an appointment; your mind being ahead of you, the body makes an over effort to keep up with it, and when you get to the desired spot, you raise your hat and find yourself in a perspiration; you take a hat, and the forehead will be moist; let the hat remain a few moments and feel the forehead again, and it will be dry, showing that the room is actually cooler than your body, and that with your out-door clothing on, you have cooled off full soon. Among the severest colds I have known men to take, were the result of sitting down to a meal in a cool room, after a walk; or being engaged in writing, have let the fire go out, and their first admonition of it was that creeping chilliness which is the ordinary forerunner of a severe cold. Persons have often lost their lives by writing or reading in a room where there was no fire, although the weather outside was rather uncomfortable. Sleeping in rooms long unused, has destroyed the life of many a visitor and friend. Our splendid parlors, and our nice "spare rooms," help to enrich many a doctor. The cold sepulchral parlors of New York, from May until November, bring disease, not only to visitors, but to the visited; for coming in from domestic occupations, or from the hurry of dressing, the heat of the body is higher than natural, and having no cloak or hat on in going in to meet a visitor, and having in addition but little vitality, in consequence of the very sedentary nature of town life, there is but very little capability of resistance, and a chill and cold is the result.

But *how to cure a cold promptly?* that is a question of life and death to multitudes. There are two methods of universal application: 1st, obtain a bottle of cough mixture, or a lot of cough candy, any kind will do; in a day or two you will *feel* better, and in high spirits; you will be charmed with the promptness of the medicine; make a mule of yourself, by giving your certificate of the valuable remedy, and in due course of time, another certificate will be made for your admission, foot foremost, into "Greenwood."

The other remedy is, consult a respectable resident physician.

---

### COTTAGE PUDDING.

One cup of butter, 2 cups sugar, 1½ cups sweet milk, 3½ cups flour, 1 teaspoonful soda and 2 of cream tartar. Flavor with vanilla.

### FRUIT PUDDING.

One teacupful of beef suet chopped fine, 1 teacupful of molasses, 1 do. of sweet milk, ½ pound raisins chopped, 1 teaspoonful soda (no cream tartar), 1 teaspoonful salt, 1 do. ground cinnamon, 1 do. cloves, ½ a nutmeg, 3 cups flour. Boil 4 hours in a tin pudding mould. The water must be boiling when put in and kept boiling until the pudding is taken out.

### SAUCE FOR ABOVE PUDDINGS.

One teacupful of butter, 2 do. pulverized sugar, beaten to a cream in an earthen dish. Then add a little less than a cup of sherry wine, stirring it in a teaspoonful at a time until the mass is blended into a light cream. Ten minutes before using put the dish in a pan of boiling water on the stove, stirring the sauce till quite hot, but not boiling.

